# Large-scale identification of small noncoding RNA with strand-specific deep sequencing and characterization of a novel virulence-related sRNA in *Brucella melitensis*

**DOI:** 10.1038/srep25123

**Published:** 2016-04-26

**Authors:** Zhijun Zhong, Xiaoyang Xu, Xinran Li, Shiwei Liu, Shuangshuang Lei, Mingjuan Yang, Jiuxuan Yu, Jiuyun Yuan, Yuehua Ke, Xinying Du, Zhoujia Wang, Zhihua Ren, Guangneng Peng, Yufei Wang, Zeliang Chen

**Affiliations:** 1 College of Veterinary Medicine, Key Laboratory of Animal Disease and Human Health of Sichuan Province, Sichuan Agricultural University, Sichuan Province, Chengdu 611130, P. R. China; 2 Institute of Disease Control and Prevention, Academy of Military Medical Sciences, Beijing 100071, P. R. China; 3 Wangjing Hospital, Academy of Traditional Chinese Medicine, Beijing 100102, P. R. China; 4 Department of Laboratory Medicine, The General Hospital of Chinese People’s Armed Police Forces, Beijing 100039, P. R. China; 5 Key Laboratory of Zoonotic of Liaoning Province, College of Animal Science and Veterinary Medicine, Shenyang Agricultural University, Liaoning Province, Shenyang 110866, P. R. China

## Abstract

*Brucella* is the causative agent of brucellosis, a worldwide epidemic zoonosis. Small noncoding RNAs (sRNAs) are important modulators of gene expression and involved in pathogenesis and stress adaptation of *Brucella*. In this study, using a strand-specific RNA deep-sequencing approach, we identified a global set of sRNAs expressed by *B. melitensis* 16M. In total, 1321 sRNAs were identified, ranging from 100 to 600 nucleotides. These sRNAs differ in their expression levels and strand and chromosomal distributions. The role of BSR0441, one of these sRNAs, in the virulence of *B. melitensis* 16M was further characterized. BSR0441 was highly induced during the infection of macrophages and mice. The deletion mutant of BSR0441 showed significantly reduced spleen colonization in the middle and late phases of infection. The expression of the BSR0441 target mRNA genes was also altered in the BSR0441 mutant strain during macrophage and mice infection, which is consistent with its reduced intracellular survival capacity. In summary, *Brucella* encodes a large number of sRNAs, which may be involved in the stress adaptation and virulence of *Brucella*. Further investigation of these regulators will extend our understanding of the *Brucella* pathogenesis mechanism and the interactions between *Brucella* and its hosts.

*Brucella* is the causative agent of brucellosis, a widely distributed zoonosis affecting a broad range of mammals and causing a severe debilitating febrile illness in humans^1^,^2^. Intracellular survival and replication are key virulence features of *Brucella*. During infection, *Brucella* invades and replicates in host phagocytes, where it is subject to hostile environmental conditions and encounters a variety of environmental stressors, including low pH, oxidative stresses, and nutrient deprivation^3^. This process requires the complex and multifactorial regulation of the gene expression associated with stress adaptation and intracellular survival. Many genes associated with intracellular trafficking and bacterial multiplications have been identified in *Brucella*. However, the complex posttranscriptional regulation and coordination of gene expression that allow *Brucella* to adapt to the harsh environment conditions of the host remain poorly understood.

Small noncoding RNAs (sRNAs), generally ranging from 50 to 300 nucleotides (nt) in length, are important posttranscriptional regulators in both eukaryotes and prokaryotes^4^,^5^. In bacteria, sRNAs predominantly function as coordinators of the adaptation processes in response to environmental changes, integrating environmental stress signals and controlling target gene expression. sRNAs usually regulate gene expression by base-pairing with their target mRNAs, altering the stability and/or translation of these targets. A large number of sRNAs have been identified in various bacteria, including *Escherichia coli*^6^,^7^, *Pseudomonas aeruginosa*^8^, *Pyrococcus abyssi*^9^, *Neisseria meningitides*^10^, human-avirulent *Yersinia pestis*^11^, and mycobacterial species^12^. Many of these sRNAs are induced by stress and are therefore associated with bacterial adaptation to the host and with bacterial virulence.

Several reports have shown that *Brucella* also encodes sRNAs. A *Brucella* deletion mutant of Hfq, a protein required for sRNA–mRNA interactions, was extremely attenuated in mice, with increased sensitivity to various environmental stresses^13^, suggesting that sRNAs have a regulatory function in the pathogen–host interactions during *Brucella* infection. Caswell *et al*. identified two sRNAs associated with the virulence of *B. abortus*, suggesting a role for sRNAs in *Brucella* pathogenicity^14^. Dong *et al*. also identified 129 candidate sRNAs in *B. abortus* 2308 with SIPHT and nucleic acid phylogenetic profiling, and found that a novel sRNA, designated BsrH, regulates the expression of the *hemH* gene^15^,^16^. In a previous study, we predicted *B. melitensis* sRNAs by bioinformatic methods and identified a novel sRNA that modulates the intracellular survival of *Brucella*^17^. Recently, Saadeh *et al*. identified 33 candidate sRNAs associated with Hfq in *B. suis*, 10 of which were confirmed with reverse transcription (RT)–PCR and/or northern blotting^18^. All these data imply that sRNAs play important roles in *Brucella*, thus identification of sRNAs associated with *Brucella* intracellular survival may provide new insights into its pathogenesis. New technologies, such as high-throughput RNA sequencing (RNA-seq) and high-density microarrays, have recently provided invaluable insights into the gene expression patterns and sRNA outputs of diverse bacteria, including several pathogens^19^,^20^,^21^. In this study, we used strand-specific deep sequencing to identify candidate sRNAs in *B. melitensis*. We also investigated the role of a novel sRNA, BSR0441, in the intracellular survival of *B. melitensis*.

## Results

### Sequencing the *B. melitensis* transcriptome

To obtain whole transcript sequences, *B. melitensis* 16M was cultured in tryptic soy broth (TSB, pH 7.0) and its total RNA was isolated. After the ribosomal RNA (rRNA) was removed, a strand-specific library was constructed with the deoxyuridine triphosphate (dUTP) second-strand marking procedure and then sequenced with the Illumina HiSeq 2000 system. After the data were filtered, 13,249,202 cDNA reads were obtained: 97.29% of the reads were mapped to the *B. melitensis* 16M genome and 65.79% were mapped to annotated genes (Figure S1); 94.24% and 63.72% of the reads were uniquely mapped to the genome and genes, respectively. This difference of 30.52% represents reads that may be located in intergenic regions. Analysis of the gene coverage showed that for 2757 (87%) genes, the mapped regions exceeded 90% of the gene length (Figure S1). The randomness of mRNA/cDNA fragmentation was evaluated from the distribution of reads in reference genes. The reads were located in relative positions in the reference genes (ratio of read location in the reference gene to the length of the reference gene). The distributions of the reads in the reference genes showed that the generated reads were randomly distributed along the genes, implying that fragmentation was homogeneously random (Figure S1).

### Identification and characteristics of candidate sRNAs

With strand-specific RNA deep sequencing, potential candidate sRNAs were identified based on the criteria of the gene model and the location between genes. In total, 1321 candidate sRNAs were identified (Table S1). The lengths of these candidate sRNAs ranged from 100 to 600 nt. The reads per kilobase of transcript per million mapped reads (RPKM) ranged from 19.1 to 9986.4, demonstrating a 522-fold difference in their expression. Of these sRNAs, 871 (65.9%) were located on chromosome I and 641 (48.5%) on the positive strand (Figure S2 and Table 1). The sRNAs density (number per Mb length) for chromosome I was 412.80, which was higher than that for chromosome II (384.62). The length distributions of the candidate sRNAs differed significantly between the two chromosomes (P < 0.01). The target gene(s) was predicted for each sRNA by TargetRNA2, and all the sRNAs had at least one potential target gene in the genome of *Brucella* (data not shown).

### Experimental verification of the novel sRNA BSR0441

**Figure 1:**
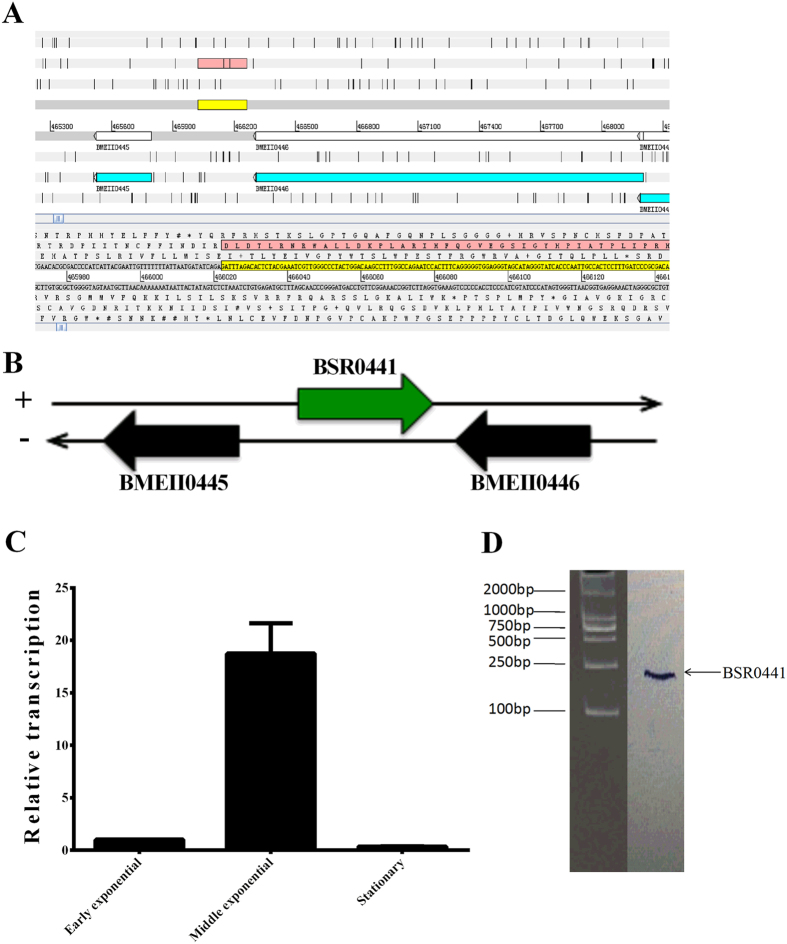
Experimental verification of BSR0441. (**A**) Location of BSR0441 (upstream and downstream genes) on the *B. melitensis* 16M chromosome; (**B**) encoded direction of BSR0441 relative to adjacent genes; (**C**) expression during different growth phases (E, exponential growth phase; M, middle growth phase; S, stationary growth phase); (**D**) northern blotting confirmation.

Because many bacterial sRNAs act as posttranscriptional regulators by base-pairing with their target mRNAs, we focused on the candidate sRNAs whose predicted target genes were involved in *Brucella* virulence. A considerable number of these candidate sRNAs have potential target genes which are involved in virulence. One of these sRNAs, candidate_1069, was chosen for further functional analysis. sRNA candidate_1069 was located downstream from BMEII0441 and upstream from BMEII0453, so the sRNA was designated BSR0441 based on the gene number of its downstream protein-coding gene. The potential target mRNA genes of BSR0441 include BMEII1007 and BMEII1118, which have been shown to be involved in *Brucella* intracellular survival and virulence (Table S2, selected potential target genes of BSR0441). The genomic location of this sRNA is shown in Fig. 1A. BSR0441 is 240 nt, encoded in a clockwise orientation at base pairs 466022–466261 on chromosome II of *B. melitensis*. The transcriptional direction of BSR0441 is opposite those of the two flanking open reading frames (ORFs), suggesting that this sRNA is transcribed independently of the flanking ORFs (Fig. 1B).

Quantitative RT–PCR (RT–qPCR) was used to verify the presence of BSR0441 in *B. melitensis* 16M. The expression level of BSR0441 was almost 20-fold higher in mid-exponential phase than in early-exponential phase or stationary phase, so the expression profile of BSR0441 is growth-phase dependent (Fig. 1C). Northern blotting hybridization was used to confirm the transcription of BSR0441 during the exponential growth phase. As shown in Fig. 1D, one band was specifically detected by the probe targeting BSR0441. All these data confirm the presence of BSR0441 in *B. melitensis* 16M.

### Expression of BSR0441 under *in vitro* stress conditions and during *in vivo* infection

**Figure 2:**
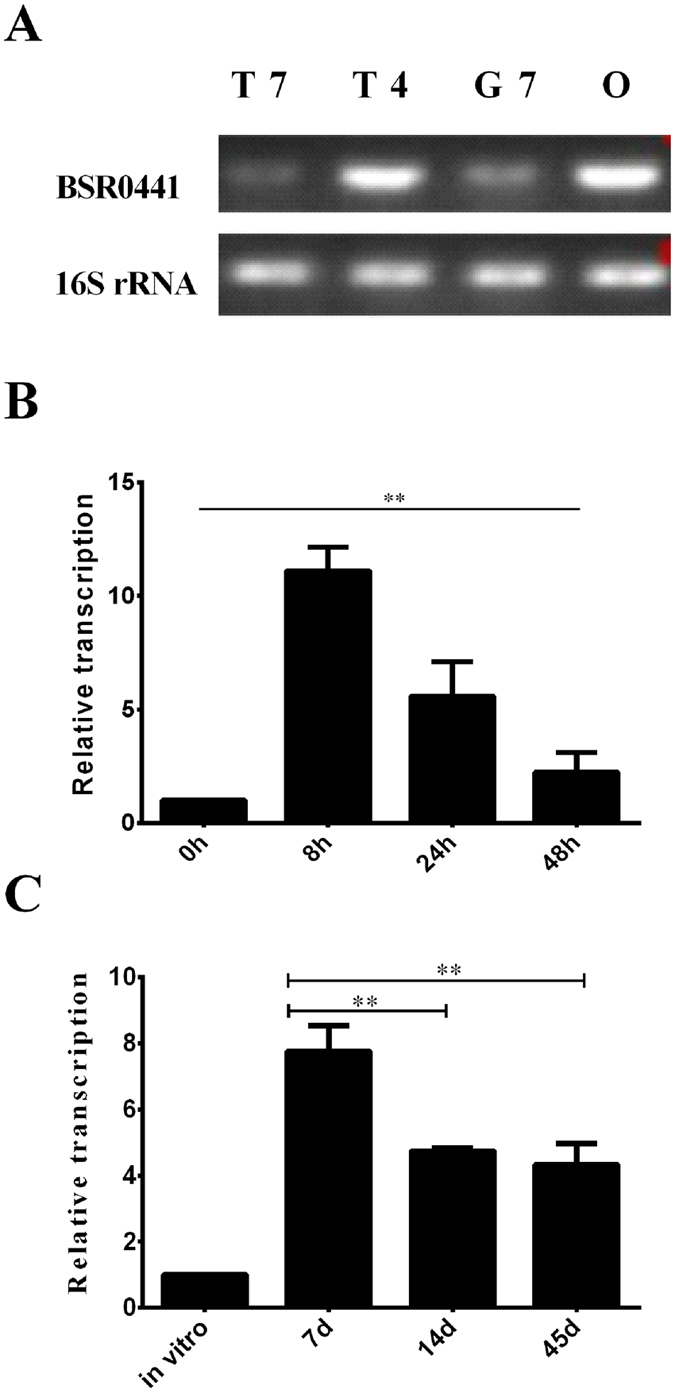
BSR0441 expression is induced or inhibited under infection-related conditions. (**A**) Induction under stress conditions; (**B**) expression during macrophage infection; (**C**) expression during mouse infection.

To understand the function of BSR0441, we monitored its expression under virulence mimicking conditions. We exposed *B. melitensis* to three different stimuli resembling those *Brucella* probably encounters during infection. As shown in Fig. 2A, compared with laboratory growth condition (T7), the expression of BSR0441 was higher at T4, G7, and O, with highest expression at T4. These observations indicate that this sRNA is expressed under stresses that simulate the conditions encountered in the host phagocytes, suggesting its possible role in *Brucella* intracellular survival. To further confirm the role of BSR0441 during intracellular infection by *B. melitensis*, the expression profile of BSR0441 during infection of macrophages and mice was determined. We first quantified the relative abundance of BSR0441 at different times after bacterial entry into murine macrophage-like RAW264.7 cells. Intracellular bacteria were collected at 0, 8, 24, and 48 h postinfection and the transcript levels of BSR0441 were measured with qRT–PCR. The levels of BSR0441 increased significantly after the infection of macrophage cells compared with those in the bacteria in the inoculum. The transcription level of BSR0441 increased approximately 12-fold by 8 h, but decreased at 24 and 48 h (Fig. 2B). Then, we isolated the total RNA from the spleens of mice infected with *B. melitensis* 16M, at different time points after inoculation. The expression of BSR0441 RNA was determined by qRT–PCR. BSR0441 was present at higher levels in the infected spleen tissues than under *in vitro* conditions . The BSR0441 levels peaked at 7 days after infection and then decreased, implying that BSR0441 functions in the early stage of mice infection. This is consistent with the results of the *in vitro* assay. Taken together, these results indicate that BSR0441 is highly activated during infection, suggesting its role during *B. melitensis* infection.

### Role of BSR0441 in the intracellular survival of *B. melitensis*

**Figure 3:**
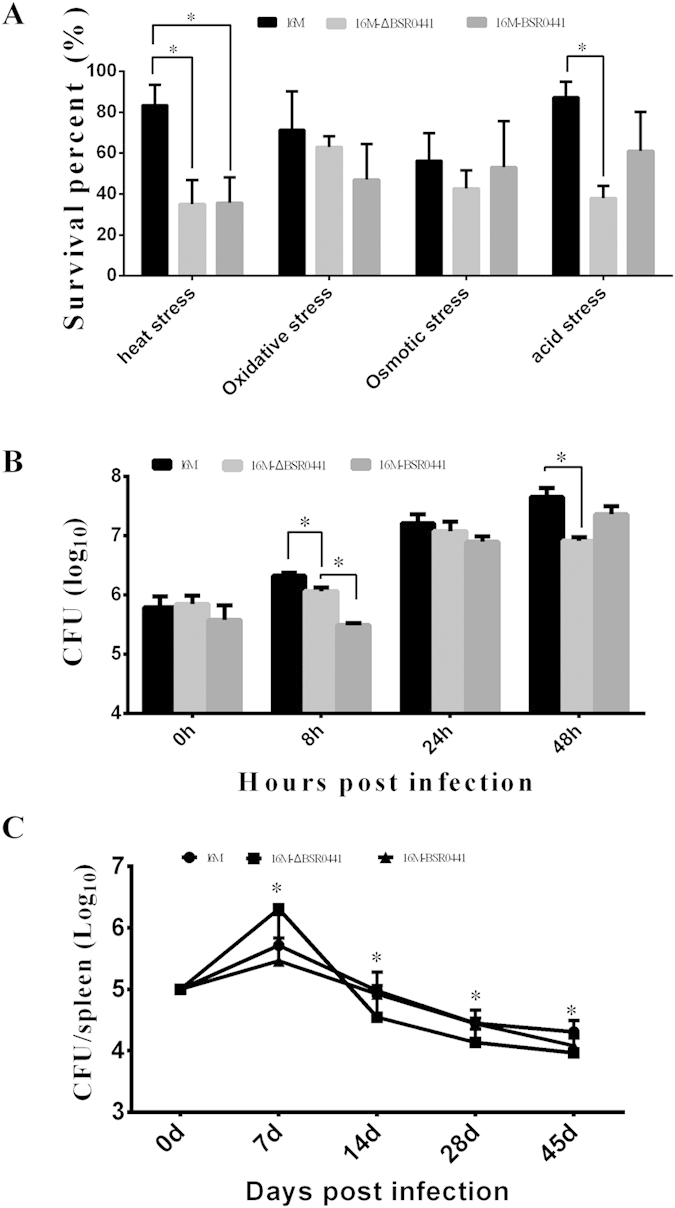
BSR0441 is involved in intracellular survival. (**A**) Survival under stress conditions. Compared with the wild-type strain 16M, the survival of the BSR0441 mutant 16MΔBSR0441 was reduced under heat and acid stress (P < 0.05). (**B**) Survival in macrophage cells. 16MΔBSR0441 showed significantly reduced survival at 8 and 48 h after infection. (**C**) Survival in mice. Compared with the wild-type strain 16M and the BSR0441-overexpressing strain 16M-BSR0441, the survival of the mutant 16MΔBSR0441 was elevated at 7 days, but decreased again at 14, 28 and 45 days.

To define the role of BSR0441 in the intracellular survival of *B. melitensis*, a BSR0441 deletion mutant (16MΔBSR0441) and a BSR0441-overexpressing strain (16M-BSR0441) were constructed. RT–PCR results confirmed that the deletion mutant did not express BSR0441 and the flanking genes of BSR0441 were not affected (data not shown). The survival capacities of 16MΔBSR0441 and 16M-BSR0441 were then evaluated under *in vitro* stress conditions and in macrophages. The survival capacity of *Brucella* was significantly reduced when BSR0441 was inactivated under heat stress or acid stress (Fig. 3A). This indicates that BSR0441 is involved in the adaptation of *Brucella* to these two stresses, which simulate the *in vivo* conditions that *Brucella* encounters in host macrophages. In macrophages, the 16MΔBSR0441 mutant was significantly reduced at 8 h and 48 h compared with the levels of strain 16M (Fig. 3B), suggesting that BSR0441 is important for the survival of *Brucella* in macrophages. To determine whether BSR0441 is important for the pathogenesis of *Brucella in vivo*, BALB/c mice were infected with the constructed BSR0441 mutants. Compared with strain 16M and 16M-BSR0441, the level of colonization by 16MΔBSR0441 increased after 7 days, but was reduced at 14, 28, and 45 days postinfection (P < 0.05) (Fig. 3C). These results imply that BSR0441 inhibits the survival of *B. melitensis* in the early stage of infection, but promotes its survival in the middle and late stages. Altogether, our data from both *in vitro* and *in vivo* assays confirmed that BSR0441 plays an important role in the intracellular survival of *Brucella*.

### BSR0441 regulates target gene expression during *Brucella* infection *in vivo*

**Figure 4:**
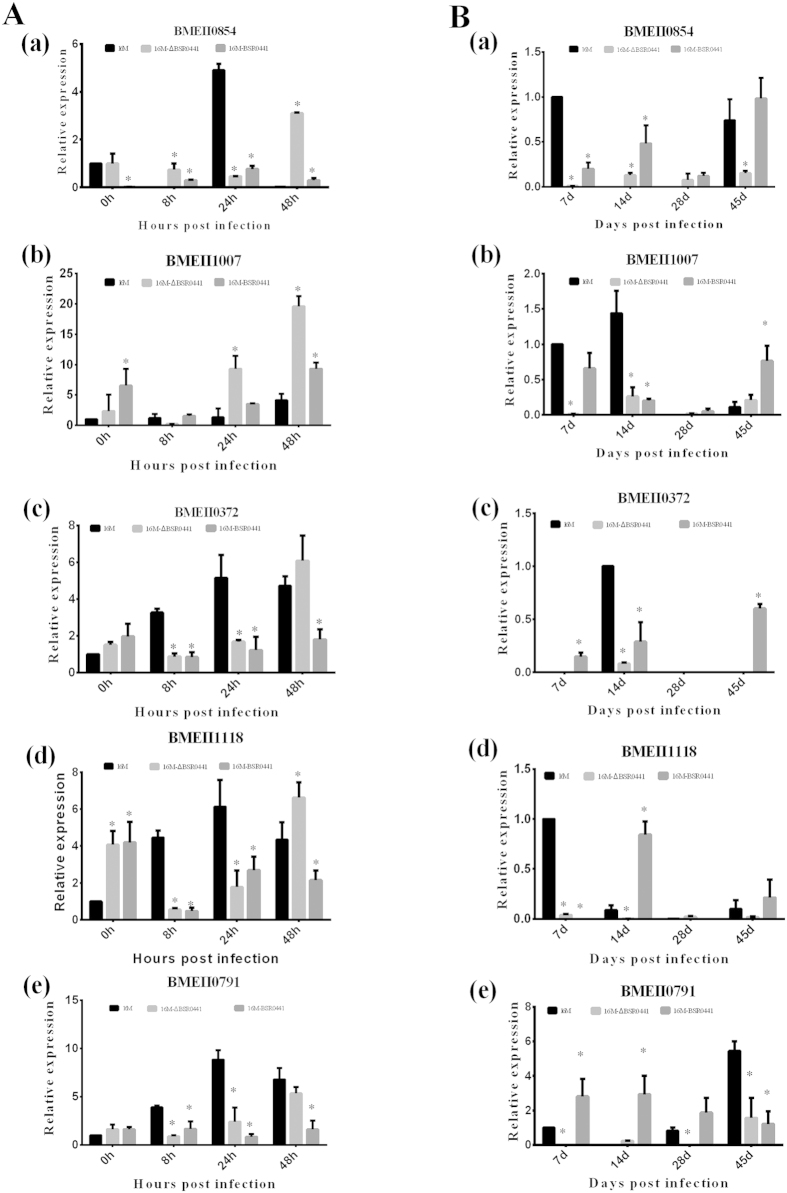
Expression of BSR0441 target genes in macrophages and during mouse infection. (**A**) Expression of BSR0441 target genes in macrophages; (**B**) expression of BSR0441 target genes in mice; (a), (b), (c), (d), and (e) present the expression profiles of target genes BMEII0854, BMEII1007, BMEII0372, BMEII1118, and BMEII0791, respectively, at predetermined times.

Many sRNAs function by affecting the expression of their target genes, so identifying its target mRNA(s) should clarify the role of BSR0441 in the intracellular survival of *Brucella*. Five putative mRNA targets of BSR0441 were predicted with TargetRNA2. As shown in Figure S3, the five target genes, BMEII0854, BMEII1007, BMEII0372, BMEII1118, and BMEII0791, have sequences homologous to BSR0441. All these five target genes are located on chromosome II and four encode transcriptional regulators associated with *Brucella* virulence. To test whether these genes are really regulated by BSR0441, qRT–PCR was performed to determine the expression of these target mRNAs in the 16M, 16MΔBSR0441, and 16M-BSR0441 *Brucella* strains isolated from infected macrophages, using the expression of 16S rRNA as the internal control. Compared with their levels in strain 16M, the expression levels of BMEII0372, BMEII1118, and BMEII0791 in 16MΔBSR0441 and 16M-BSR0441 were downregulated at 8 and 24 h postinfection (P < 0.05). The levels of BMEII1007 showed no apparent change in 16MΔBSR0441, except that at 24 h (P < 0.05) (Fig. 4A). These data indicate that BSR0441 affects the expression of its target genes in macrophages.

The expressions of the five target genes were also analyzed *in vivo* during the infection of mice by *Brucella*. As shown in Fig. 4B, the expression of BMEII0791, BMEII1118, BMEII1007, and BMEII0854 in strain 16MΔBSR0441 was repressed at 7 day post infection. However, on day 28, the expression profiles of the target genes showed no apparent change, except for BMEII0791, which was significantly reduced in 16MΔBSR0441. All five target genes were significantly differentially expressed in 16MΔBSR0441 on days 7 and 14 compared with their expression in the parent strain 16M, indicating that BSR0441 affects the expression of its target genes during the early phase of *Brucella* infection *in vivo*.

## Discussion

Clarification of *Brucella* intracellular survival mechanism in harsh environments is critical for understanding its pathogenesis. Previous studies have shown that sRNAs play important roles in the intracellular survival of *Brucella*^14^,^17^. As a convenient and effective tool for transcriptome studies, RNA-seq has facilitated the genome-wide detection of expressed sRNAs in bacterial pathogens. Recently, using deep sequencing, Saadeh *et al*. identified 33 candidate sRNAs associated with Hfq in *B. suis*^18^. Therefore, we used a strand-specific deep RNA sequencing strategy to identify the sRNAs in *B. melitensis* 16M. In total, 1321 candidate sRNAs were successfully identified. Thus, a large number of sRNAs were identified in the *Brucella* chromosomes compared with the number of genes in this pathogen. The genome of *Brucella* contains about 3.2 Mb, and 3200 coding genes have been annotated. The 1321 candidate sRNAs are equivalent to 41.3% of the whole gene number, indicating that sRNAs are common in *Brucella*. Of the cDNA reads generated, 94.24% mapped uniquely to genomic sequences, but only 63.72% mapped to the annotated genes. Therefore, 30.52% of the reads mapped to intergenic regions, indicating that intergenic regions are actively transcribed in *Brucella*. Gene models were identified in the mapped intergenic regions and novel actively transcribed regions identified. Small ORFs were also identified in these regions (unpublished data). These results show that sRNAs exist in the intergenic regions of the *Brucella* genome and may play important roles. The unexpectedly large number of sRNAs also suggests that *Brucella* fine-tunes its metabolism at the posttranscriptional level with RNA regulators during intracellular infection.

The *Brucella* genome contains two chromosomes: chromosome I is about 2.1 Mb and chromosome II is 1.1 Mb. The genes located on chromosome I are mainly housekeeping genes, whereas those on chromosome II are involved in virulence and environmental adaptation^22^. Some researchers consider that chromosome II is a large plasmid of *Brucella*^23^. We compared the distributions of sRNAs on the two chromosomes. Interestingly, the density of sRNAs was higher on chromosome I than on chromosome II, although whether there are more sRNAs on chromosome II remains to be determined. In this study, only one culture condition was used, and it is possible that many other sRNAs are not expressed under this condition. The mean RPKM value for all the identified candidate sRNAs was 460.8, whereas the RPKM values for individual sRNAs ranged from 19 to 9986, indicating that the expression levels of the identified sRNAs differed significantly. This indirectly implies that these sRNAs are differentially required under this condition.

To clarify the importance of these candidate sRNAs, one of them, BSR0441, was chosen for further functional analysis because its predicted target genes are involved in the intracellular survival of *Brucella*. Because gene induction is closely related to the function of the encoded protein, the expression profile of BSR0441 was analyzed both under *in vitro* stresses and during *in vivo* infection. As expected, the expression of BSR0441 was induced by stresses mimicking those encountered in host cell, indicating that BSR0441 is associated with the adaptation of the bacterium to its host, which has also been observed in other pathogens^24^. Furthermore, the expression levels of BSR0441 varied with the stresses applied and the stage of infection, implying that BSR0441 acts in specific infection stages. Therefore, the expression profile of BSR0441 was closely related to the survival capacity of *Brucella* under stress. BSR0441 was strongly expressed at 8 h during macrophage infection and 7 days of mouse infection, indicating that it functions in an early stage of infection. Consistent with this expression profile, the survival capacity of the BSR0441 mutant was also significantly reduced in the early stage of macrophage infection. Splenic bacterial counts were higher in the 16MΔBSR0441-infected mice than in mice infected with the wild-type strain 16M at 7 days postinfection. However, the survival capacity of the BSR0441 mutant decreased after this time point, implying that BSR0441 is important for the repression of *Brucella* survival in an early stage of mice infection, which is essential for the establishment of a replication niche to allow chronic infection.

A number of mRNAs encoding transcription regulators appear to be targets of multiple sRNAs^25^,^26^. Therefore, the identification of target mRNAs may clarify the roles of sRNAs in the intracellular survival of *Brucella*. In this study, five putative mRNA targets of BSR0441 were predicted with TargetRNA2. According to the Clusters of Orthologous Groups functional classification, the five target genes encoded a transcriptional regulator of the MerR family (BMEII0372), a two-component response regulator (BMEII0791), a transcriptional regulator of the CRP family (BMEII0854), a transcriptional regulator of the GntR family (BMEII1007), and a multidrug-resistance protein A (BMEII1118). Four of them (BMEII0372, BMEII0791, BMEII0854, and BMEII1007) are transcriptional regulators associated with *Brucella* virulence. BMEII0854, which is a CRP family transcriptional regulator, responds to various environmental stimuli (temperature, pH, etc.) and subsequently triggers the expression of many stress-response and virulence-related genes^27^. In a previous study, we demonstrated that BMEII0854 is regulated by Hfq. The expression of BMEII0854 mRNA was lower in an *hfq* mutant strain of *Brucella* than in the wild-type strain 16M^28^. In the present study, the expression of BMEII0854 was also affected by BSR0441. BMEII0854 mRNA was significantly lower in 16MΔBSR0441 at 7 days postinfection than in the parental strain 16M (Fig. 4B). Hfq is a chaperone protein that promotes base-pairing interactions between sRNAs and its target mRNA^29^,^30^,^31^. A recent study suggested that **s**RNAs associated with *Hfq* are related to virulence or stress adaptation in *B. suis*^18^. Therefore, we inferred that Hfq may paly role in the interaction between BSR0441 and BMEII0854. It will be interesting to test the detailed Hfq-mediated regulation mechanism of BSR0441 on BMEII0854. There is little information on the roles of the other four target genes of BSR0441 identified in this study in the intracellular survival of *Brucella*. However, by analyzing homologous genes, we found that three of these four target genes are related to the virulence of the pathogen, but their functions in the intracellular survival of *Brucella* require further study.

In summary, using strand-specific deep sequencing, we identified 1321 candidate sRNAs in *B. melitensis*. These sRNAs are differentially distributed on the two bacterial chromosomes. Although a large number of sRNAs were identified, more unidentified sRNAs may exist because only one culture condition was examined in this study. Analysis of the newly identified sRNA BSR0441 indicated that it is responsible for the bacterial adaptation to stress conditions and contributes to the intracellular survival of *Brucella*. Future work will characterize these sRNA regulators further and define their roles in the stress adaptation and virulence of *Brucella*.

## Materials and Methods

### Ethics statement

Female 6–8-week-old BALB/c mice were obtained from the Animal Center of Military Medical Sciences. All experimental methods and animals care performed in the present study were approved accordance with the guidelines of the Experimental Animal Regulation Ordinances defined by the China National Science and Technology Commission, and the animal work was approved by the animal ethics committee of the Beijing Institute of Disease Control and Prevention (Ethical Approval BIDCP008-2014). The animals were provided with humane care and healthful conditions during their stay in the facility. All individuals who handled the animals received instructions in experimental methods and in the care, maintenance, and handling of mice, and were under the committee’s supervision.

### Bacterial strains, plasmids, and primers

All *Brucella* strains used were derivatives of *B. melitensis* 16M. The *Brucella* strains were maintained on tryptic soy agar (TSA) plates and routinely cultured at 37 °C in TSB, whereas the other strains were maintained in and/or grown on Luria–Bertani (LB) medium. When necessary, antibiotics were added into the medium at the following concentrations: kanamycin at 50 μg/ml and ampicillin at 100 μg/ml. The plasmid pBBR1MCS4 contains the ampicillin-resistance gene (*ampR*) and can replicate in *Brucella*^32^. The primers used in this study are listed in Table S3.

### Strand-specific dUTP library preparation for Illumina sequencing

For deep sequencing, total RNA was prepared from *B. melitensis* 16M cultures grown in 100 ml of liquid TSB medium. RNAprotect Bacterial Reagent (Qiagen, Valencia, CA) was added to the cultures grown to mid-exponential phase, according to the manufacturer’s instructions. The treated cells were stored at −80 °C (for no longer than 24 h) until RNA isolation. The total RNA was isolated and purified with the MasterPure™ RNA Purification Kit (Epicenter), according to the manufacturer’s instructions. RNA quality was determined with Bioanalyzer (Agilent Technologies) and quantified with a ND-1000 spectrophotometer (NanoDrop Technologies). Total RNA samples (10 μg) were subjected to further purification by enriching them in mRNA with the MICROBExpress Kit (Ambion). The samples were resuspended in 15 μL of RNase-free water. Strand-specific libraries were prepared using a dUTP second-strand marking protocol with reagents from Invitrogen, unless otherwise stated^33^,^34^. The first-strand cDNA was synthesized from 400 ng of precipitated fragmented RNA, using 3 μg of random primers, 4 μg of actinomycin D, and Superscript III Reverse Transcriptase. After the extraction and precipitation of the first strand, the second-strand cDNA was synthesized using dUTP rather than dTTP, as previously described^34^. Paired-end libraries for Illumina sequencing were prepared from the purified cDNA (MinElute PCR Purification Kit; Qiagen), as recommended by Illumina HiSeq 2000.

### Small noncoding RNA identification

We first identified transcriptionally active regions longer than 100 bp and with an average coverage depth >2. Gene models found in the intergenic regions (100 bp from the upstream and downstream genes) were considered candidate sRNAs. These candidate sRNAs were then screened against the sRNAMap, sRNATarBase, and SIPHI databases based on the similarity of their sequences and against the Rfam database based on consensus secondary structures. The potential target genes of sRNAs were predicted with TargetRNA2^35^.

### RT–PCR

For RT–PCR, northern blotting, and qRT–PCR analyses, total RNA was isolated from liquid *B. melitensis* cultures with TRIzol Reagent (Invitrogen), according to the manufacturer’s protocol. The samples were treated with DNase I to remove any residual DNA. The quantity and purity of the RNA was determined with a NanoDrop ND-1000 spectrophotometer (Thermo Scientific). Samples of the extracted RNA (2 μg) were reverse transcribed to cDNA with the ImProm-II™ Reverse Transcription System (Promega). PCRs were then performed in a reaction system comprising 12.5 μl of 2 × PCR Reagent (TianGen), 1 μl of each primer, 1 μl of cDNA as the template, and ddH_2_O to a final volume of 25 μl. The thermocycling conditions were: 5 min at 94 °C for predenaturation, and then 35 cycles of amplification (30 s at 94 °C, 30 s at 55 °C, 30 s at 72 °C), and 5 min at 72 °C. The amplification product was separated electrophoretically in 1% agarose gel containing ethidium bromide, and detected with the Gel Doc™ XR System (Bio-Rad Laboratories).

### Northern blotting

Northern blot analyses were performed with a DIG Northern Starter Kit (Roche), as previously described by Beckmann *et al*.^36^. Briefly, total RNA (20 μg/sample) was denatured at 70 °C for 5 min, run on a 10% polyacrylamide–7 M urea gel, and then transferred to Hybond N^+^ membrane (GE Healthcare) with a Mini Trans-Blot Cell apparatus (Bio-Rad Laboratories). The membrane was prehybridized in ULTRAhyb® Ultrasensitive Hybridization Buffer (Ambion) for 45 min, and 3′-end DIG-labeled RNA probes were added. The membranes were then hybridized overnight at 64 °C in DIG Easy Hyb, according to the manufacturer’s protocol.

### Quantitative RT–PCR

Quantitative RT–PCR (qRT-PCR) was performed with the IQ^5^ Real Time PCR Detection System (Bio-Rad Laboratories) with SYBR Premix Ex Taq™ (Tli RNaseH Plus) (Takara Biochemicals) in a 25-μl volume containing 12.5 μl of Ex Taq mixture, 0.6 μl of each primer, 1 μl of cDNA as the template, and ddH_2_O, with the following thermocycling conditions: 95 °C for 30 s, followed by 40 cycles of amplification (30 s at 95 °C, 30 s at 60 °C). The relative transcription levels of the target genes were determined with the 2^–ΔΔCt^ method^37^, and normalized to 16S rRNA. Each assay was performed in triplicate.

### Construction of mutant and overexpressing strains

To generate the deletion strain of BSR0441, a suicide plasmid designated pUC19K-BSR0441 was constructed by assembling into pUC19K fragments of approximately 500 bp from the regions upstream and downstream from the BSR0441 coding region^38^. Competent *B. melitensis* 16 M cells were then transformed with this plasmid with the Bio-Rad MicroPulser. The potential deletion mutants were screened based on their amp^S^ kan^R^ phenotype and identified with PCR using primer sets pUC19K-F/BSR0441-I-R and pUC19K-R/BSR0441-I-F. The BSR0441-overexpressing strain of *Brucella* was constructed with a method previously described by Cui *et al*.^28^. The fragments of the BSR0441 locus were amplified from the *B. melitensis* 16M genome with the primer set BSR0441-N’-F and BSR0441-C’-R and cloned into the *Spe*I–*Xho*I sites of pBBR1MCS4, a plasmid containing the *amp*^*R*^ gene that can replicate in *Brucella*. The wild-type strain 16M was then transformed with the resulting plasmid and the recombinant strain was confirmed with PCR and DNA sequencing. The deletion and overexpression of BSR0441 were confirmed with RT–PCR.

### Determination of BSR0441 expression under *in vitro* stress conditions

We exposed *B. melitensis* to three different stimuli resembling those *Brucella* probably encounters during infection: low pH (TSB medium at PH4.0) (TSB 4.0; T4), limited nutrition (GEM medium, MgSO_4_•7H_2_O 0.2 g/L, Citric acid H_2_O 2.0 g/L, K_2_HPO_4_ 10.0 g/L, NaNH_4_HPO_4_•4H_2_O 3.5 g/L, Glucose 20 g/L, pH 7.0) (GEM 7.0; G7), and oxidative stress (induced with H_2_O_2_; O)^39^. TSB 7.0 (T7) is the standard *in vitro* growth condition for *B. melitensis*. The expression of BSR0441 was examined with RT–PCR, using 16SrRNA as the internal control.

### Stress resistance assays

All the tested *B. melitensi*s strains were cultured in TSB medium to the stationary growth phase (OD_600_ = 2.5) at 37 °C. To assay the effects of high-osmolarity stress, acidification, H_2_O_2_, and heat treatment, the cells were incubated in the presence of NaCl (1.5 M) for 20 min, in TSB medium at pH 3.0 for 15 min, in the presence of 440 mM H_2_O_2_ for 40 min, or at 50 °C for 60 min, respectively. The cells were 10-fold serially diluted and spread on TSA plates to determine the numbers of viable bacteria. Each experiment was performed in triplicate.

### Macrophage survival assay

Murine macrophage-like RAW264.7 cells were used to assess the survival capacity of *Brucella* strains 16M, 16MΔBSR0441, and 16M-BSR0441. In brief, monolayers of macrophages were seeded in 24-well plates at a density of 5 × 10^5^ cells per well and incubated for 16 h. The macrophages were infected with a bacterial suspension at a multiplicity of infection (MOI) of 50 and incubated at 37 °C under 5% CO_2_ for 45 min. The cells were washed three times with phosphate-buffered saline (PBS) and incubated with 50 μg/ml gentamycin for 60 min to kill any extracellular bacteria. The medium was then replaced with DMEM/FCS containing 25 μg/ml gentamycin. At 0, 8, 24, and 48 h postinfection, the supernatant was discarded, the cells were lysed with 1% Triton X-100, and the intracellular bacteria were plated in duplicate on TSA plates with or without kanamycin or ampicillin and counted after 3–5 days. The data are expressed as the mean log_10_CFU of three wells. An independent-samples *t* test was used to analyze the differences between the means of the groups. P values < 0.05 were considered statistically significant.

### Mouse infection

Groups of 6–8-week-old female BALB/c mice (n = 15 per group) were infected intraperitoneally with 200 μl of bacterial suspension (5 × 10^5^ CFU/ml) of strain 16M, 16MΔBSR0441, or 16M-BSR0441. At 7, 14, 28, and 45 days postinoculation, the infected mice were killed by cervical dislocation. Their spleens were removed aseptically and homogenized with PBS containing 0.1% Triton X-100. Serial dilutions of the spleen homogenates were prepared and plated in duplicate on TSA plates with or without kanamycin or ampicillin, and the colonies were counted after incubation for 3–5 days at 37 °C. The data are presented as the mean log_10_CFU values from three mice. Differences were considered significant at P < 0.05.

## Additional Information

**How to cite this article**: Zhong, Z. *et al*. Large-scale identification of small noncoding RNA with strand-specific deep sequencing and characterization of a novel virulence-related sRNA in *Brucella melitensis*. *Sci. Rep*. **6**, 25123; doi: 10.1038/srep25123 (2016).

## Supplementary Material

Supplementary Information

Supplementary Data 1

## Figures and Tables

**Table 1:** Overview of candidate sRNAs.

		Length of sRNA					
Characteristics		Average	100–200	201–300	301–400	401–500	>500
Number	I	871	152	235	206	157	121
	II	450	80	137	111	73	49
RPKM	I	458.08	361.17	408	488	519	544
	II	466.13	337	368	537	622	553
sRNA density (Number/Mb)	I	412.80	72.38	111.90	98.10	74.76	57.62
	II	384.62	72.73	124.55	100.91	66.36	44.55
Reads Density (Reads per bp)	I	33.21	26.46	30.15	35.41	35.92	40.32
	II	33.17	24.07	28.42	39.13	39.98	37.7
