# Supplementary material for: Large-scale identification of small noncoding RNA with strand-specific deep sequencing and characterization of a novel virulence-related sRNA in Brucella melitensis
Source: Sci Rep. 2016 Apr 26;6:25123. doi: 10.1038/srep25123 (PMC4845025; doi:10.1038/srep25123)
Supplement: Supplementary Information [file srep25123-s1.pdf]

Large-scale identification of small noncoding RNA with strand-specific deep sequencing and characterization of a novel virulence-related sRNA in Brucella melitensis

Zhijun Zhong[1,2#], Xiaoyang Xu[1,2#], Xinran Li[2,#], Shiwei Liu[3,#], Shuangshuang Lei[1,2], Mingjuan Yang[2], Jiuxuan Yu[2], Jiuyun Yuan[2], Yuehua Ke[2], Xinying Du[2], Zhoujia Wang[2], Zhihua Ren[1], Guangneng Peng[1,*], Yufei Wang[2,4,*], Zeliang Chen[2,5,*]

1 College of Veterinary Medicine, Key Laboratory of Animal Disease and Human Health of Sichuan Province, Sichuan Agricultural University, Sichuan Province, Chengdu 611130, P. R. China

2 Institute of Disease Control and Prevention, Academy of Military Medical Sciences, Beijing 100071, P. R. China

3 Wangjing Hospital, Academy of Traditional Chinese Medicine, Beijing 100102, P. R. China

4 Department of Laboratory Medicine, The General Hospital of Chinese People's Armed Police Forces, Beijing 100039, P. R. China

5 Key Laboratory of Zoonotic of Liaoning Province, College of Animal Science and Veterinary Medicine, Shenyang Agricultural University, Liaoning Province, Shenyang 110866, P. R. China

# These authors contributed equally to this work

* Corresponding authors:

Guangneng Peng: No. 211, Huimin Road, Wenjiang District, Chengdu 611130, P.R. China. Tel: 86-028-862-91176; Fax: +86-028-862-91176; E-mail: pgn.sicau@163.com

Yufei Wang: No. 69, Yongding Road, Haidian District, Beijing 100039, P.R. China.

Tel: 86-10-57976754; Fax: 86-10-57976754; E-mail: tosya@163.com

Zeliang Chen: No. 20, Dongdajie, Fengtai District, Beijing 100071, P.R. China.

Tel: 86-10-66948387; Fax: 86-10-66948387; E-mail: zeliangchen@yahoo.com

Supplementary material

Figures

A

Mapping Statistics	Map to Genome		Map to Gene	
	Reads number	Percentage	Reads number	Percentage
Total Reads	13249202	100.00%	13249202	100.00%
Total BasePairs	1192428180	100.00%	1192428180	100.00%
Total Mapped Reads	12889855	97.29%	8717156	65.79%
Perfect match	10471858	79.04%	7083540	53.46%
<=5bp mismatch	2417997	18.25%	1633616	12.33%
Unique match	12485618	94.24%	8442202	63.72%
Multi-position match	404237	3.05%	274954	2.08%
Total Unmapped Reads	359347	2.71%	4532046	34.21%

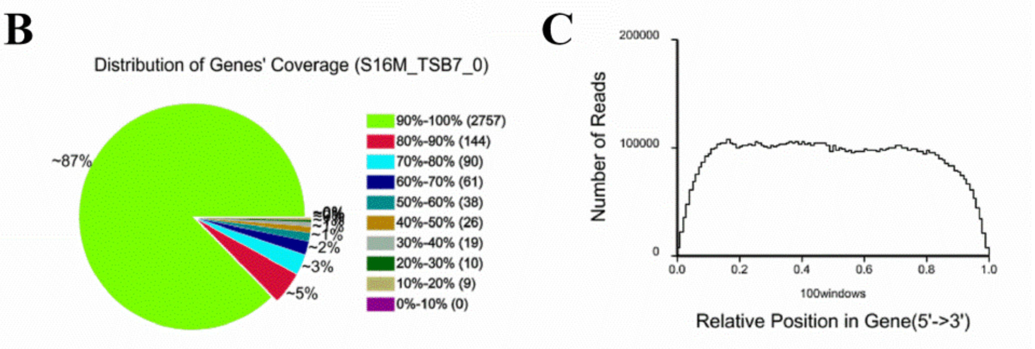

Figure S1. Statistical analysis of strand-specific RNA-seq.

(A) Mapping statistics of RNA sequencing reads; (B) distribution of gene coverage; (C) relative positions of mapped reads in genes (5′-3′).

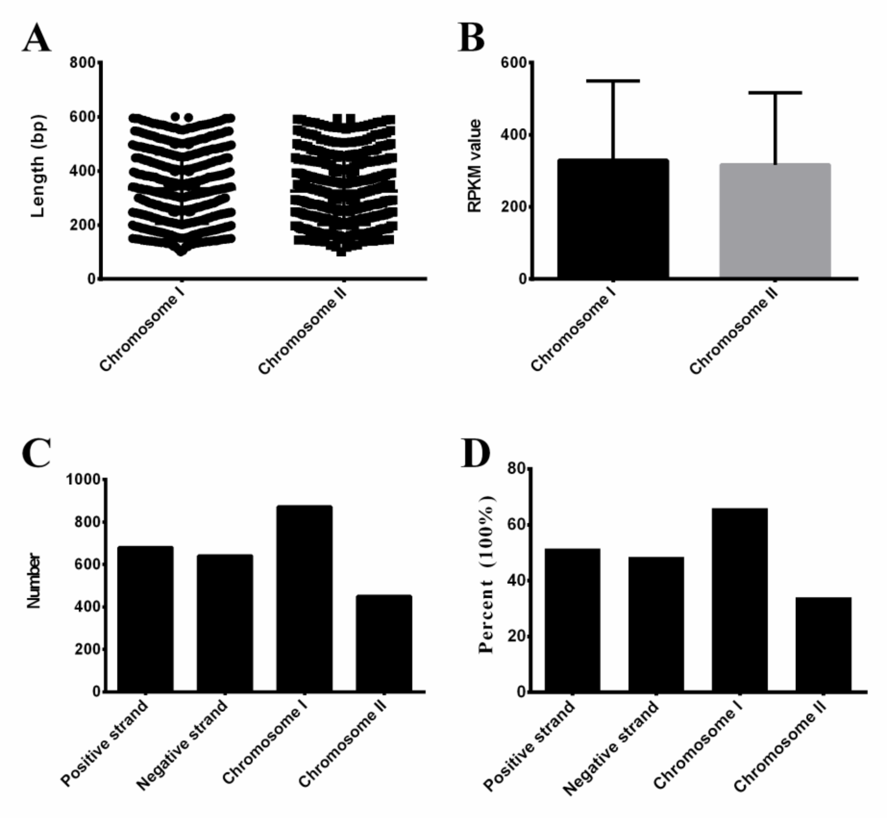

Figure S2. Distributions of candidate sRNAs.

```
BSR0441      233    3’ GUCUGCGGGUGC 5’        222
                        ||||||||||
BMEII0791    8      5’ AAGACGCCCACA 3’        19
BSR0441      218    3’ AGGGCGGGGUC 5’         208
                        :|||||||:
BMEII1118    3      5’ GUCCGCCCCGA 3’         13
BSR0441      218    3’ AGGGCGGGGUC 5’         208
                        :||||||||
BMEII0854    11     5’ AUCCGCCCCA- 3’         20
BSR0441      178    3’ GCCGC-CCCACGCUACG 5’   163
                       || |  ||||||||||
BMEII0372    -13    5’ AGGGGAGGGUGCGAUGA 3’   4
BSR0441      192    3’ CUACCCCCCUCCAG 5’      179
                       || |||||  |||
BMEII1007    -48    5’ AAU-GGGGGCGGUA 3’      -36
```

Figure S3. Predicted target genes of BSR0441.

Tables

Table S1:List of candidate sRNAs.

Table S2: Putative mRNA targets of BSR0441 identified by TargetRNA2

Locus	NCBI GI	Protein Description	COG[a]	Location[b]	Energy	Pvalue
BMEII0013	17988357	para-aminobenzoate synthase	EH	Unknown	-8.8	0.041
BMEII0047	17988391	lysophospholipase l2	I	CytoplasmicMembrane	-10.91	0.014
BMEII0051	17988395	nodulation protein w	T	Cytoplasmic	-8.59	0.044
BMEII0121	17988465	high-affinity branched-chain amino acid transport system permease protein livh	E	CytoplasmicMembrane	-15.18	0.001
BMEII0143	17988487	transcriptional regulator, arac family	-	Cytoplasmic	-10.45	0.019
BMEII0196	17988540	spermidine/putrescine-binding periplasmic protein	E	Periplasmic	-10.6	0.017
BMEII0220	17988564	oligopeptide transport system permease protein oppb	EP	CytoplasmicMembrane	-8.36	0.049
BMEII0240	17988584	dioxygenase	R	Unknown	-14.09	0.002
BMEII0305	17988650	nitrate reductase	R	Unknown	-11.13	0.013
BMEII0336	17988681	ABC transporter permease protein	P	CytoplasmicMembrane	-15.21	0.001
BMEII0349	17988694	general l-amino acid-binding periplasmic protein	ET	Periplasmic	-18.81	0
BMEII0354	17988699	sugar transporter	S	Cytoplasmic	-10.61	0.017
BMEII0369	17988714	atrazine chlorohydrolase	FR	Cytoplasmic	-9.96	0.024
BMEII0372	17988717	transcriptional regulator, merr family	K	Unknown	-17.05	0
BMEII0378	17988723	formate dehydrogenase alpha chain	C	Periplasmic	-9.41	0.031
BMEII0379	17988724	hypothetical protein	-	Cytoplasmic	-10.61	0.017
BMEII0399	17988744	hypothetical protein	-	Unknown	-11.65	0.009
BMEII0449	17988794	type i restriction-modification system restriction subunit	V	Cytoplasmic	-8.69	0.042
BMEII0476	17988821	uronate isomerase	G	Unknown	-8.43	0.047
BMEII0497	17988842	enoyl-CoA hydratase	I	Unknown	-16.73	0

BMEII0511	17988856	phosphogluconate dehydratase	-	Cytoplasmic	-10.45	0.019
BMEII0529	17988874	surface protein	S	Cytoplasmic	-14.28	0.001
BMEII0539	17988884	hypothetical cytosolic protein	S	Unknown	-10.01	0.023
BMEII0573	17988918	transcriptional regulator, rpir family	K	CytoplasmicMembrane	-8.55	0.045
BMEII0626	17988971	membrane dipeptidase	E	Unknown	-9.67	0.027
BMEII0703	17989048	fe-s oxidoreductase	J	Cytoplasmic	-9.02	0.037
BMEII0720	17989065	transcriptional regulator	-	Unknown	-15.84	0
BMEII0764	17989109	hypothetical protein	-	Unknown	-10.08	0.022
BMEII0785	17989130	transcriptional regulator, marr family	K	Unknown	-14.36	0.001
BMEII0791	17989136	two component response regulator	TK	Cytoplasmic	-19.38	0
BMEII0802	17989147	daunorubicin resistance ATP-binding protein drra	V	Cytoplasmic	-10.38	0.019
BMEII0854	17989199	transcription regulator, crp family	T	Cytoplasmic	-13.05	0.004
BMEII0917	17989262	hypothetical protein	-	Unknown	-9.57	0.029
BMEII0924	17989269	hypothetical protein	-	Unknown	-8.32	0.049
BMEII0938	17989283	myo-inositol 2-dehydrogenase	R	Cytoplasmic	-9.41	0.031
BMEII0946	17989291	laci-family transcription regulator	K	Cytoplasmic	-18.77	0
BMEII0948	17989293	nitrite extrusion protein	P	CytoplasmicMembrane	-15.08	0.001
BMEII0998	17989343	nitric-oxide reductase subunit b	P	CytoplasmicMembrane	-9.19	0.034
BMEII1007	17989352	transcriptional regulator, gntr family	K	Cytoplasmic	-8.8	0.04
BMEII1017	17989362	mercuric resistance operon regulatory protein	K	Cytoplasmic	-9.45	0.03
BMEII1021	17989366	enoyl-CoA hydratase	I	Cytoplasmic	-10.87	0.015
BMEII1040	17989385	cytosine deaminase	FJ	Cytoplasmic	-11.43	0.011
BMEII1050	17989395	hypothetical protein	-	Unknown	-8.78	0.041
BMEII1067	17989412	hypothetical cytosolic protein	S	Cytoplasmic	-10.9	0.015
BMEII1068	17989413	cytochrome c2 precursor	C	Periplasmic	-14.12	0.002
BMEII1083	17989428	hypothetical protein	S	Unknown	-9.29	0.033
BMEII1093	17989438	glycerol-3-phosphate regulon repressor	KJ	Unknown	-9.7	0.027

BMEII1118	17989463	multidrug resistance protein a	V	CytoplasmicMembrane	-13.66	0.002
BMEII1134	17989479	amidase	-	Unknown	-9.98	0.024

a: Abbreviation of cellular role categories of theoretical (http://www.ncbi.nlm.gov/COG/).

b: Abbreviation of cellular location. Protein cellular location was annotated by PSORTb V. 2.0 (http://www.psort.org/).

Table S3: Primers used in this study.

Primer names	Sequence(5'-3')
16S rRNA-F	ACTAAGGGCGAGGGTTGC
16S rRNA-R	CACTGGACCATTACTGACGC
BSR0441-RT-F	TAGACACTCTACGAAATC
BSR0441-RT-R	ACTTTAGGCTTCTAGTAG
BSR0441-p-F	GATTTAGACACTCTACG
BSR0441-p-R	TAATACGACTCACTATAGGGGGACATCCAGACGCCCACG
BSR0441-N-F	TGGACTTTCGATATGGTGCTG
BSR0441-N-R	GACATTCATCCCAGGTGGCAGTGTCTAAATCTCTGATATC
BSR0441-C-F	TCTGGGGTTCGAAATGACCGCCTACTAATCGGCATCGCAC
BSR0441-C-R	GTGACTGCGTAAGCGCTCTC
BSR0441-I-F	AGCATCGACAACAGCATGCAG
BSR0441-I-R	TCAGCATGAAAGTATGCGCAG
Kana-F	GCCACCTGGGATGAATGTC
Kana-R	TCTGGGGTTCGAAATGACCG
Kana-I-F	ATCAGGACATAGCGTTGG
Kana-I-R	GGCAAGAAAGCCATCCAG
BSR0441-N'-F	CTCGAGGATTTAGACACTCTACG
BSR0441-C'-R	ACTAGTGGACATCCAGACGCCCACG
BMEII0791-F	GGATATACGCGACCTTCTGC
BMEII0791-R	TGGCCGTCAGCATAATGATA
BMEII1118-F	TTCGACAGACGATGCCTATG
BMEII1118-R	GGATGAGCGTTTCTTTTTGC
BMEII0854-F	AACGGATCTTTCCGTTGTTG
BMEII0854-R	TTGGCTTTTGTCCTGAAACC
BMEII0372-F	GCGCATTGAAGCGTATTCTC
BMEII0372-R	TACCGCTCCTTCAGGATTTG
BMEII1007-F	ATGGAGCGTAAACGTGGTTC
BMEII1007-R	CGACTAGACCTTCGCTCGAC
